# Effect of Gradient Layer Induced by Laser Shock Peening on Adhesion and Wear Resistance of AlCrN Coatings on TC4 Titanium Alloy

**DOI:** 10.3390/ma19030608

**Published:** 2026-02-04

**Authors:** Ying Xu, Wenqian Yu, Xinlong Liao, Yuxuan Zhu, Boyong Su

**Affiliations:** 1 Engineering Training Center, Nantong University, Nantong 226019, China; 2 School of Mechanical Engineering, Nantong University, Nantong 226019, China; 3 School of Electromechanical Engineering, Guangdong University of Technology, Guangzhou 510006, China; lalgdut@163.com; 4 Faculty of Science and Technology, University of Macau, Macau 999078, China; dc32707@umac.mo

**Keywords:** TC4 titanium alloy, AlCrN coating, laser shock peening, element diffusion, gradient coating

## Abstract

To address the inherent defects in the fabrication of AlCrN titanium alloy coatings and enhance interfacial bonding strength as well as tribological performance, an AlCrN coating was employed as an absorption layer and subjected to laser shock processing to form an AlCrN/TC4 transition layer. Subsequently, a secondary AlCrN coating was deposited to construct a gradient coating architecture. The surface and cross-sectional morphologies and elemental distributions under varying laser energies were systematically investigated, and the influence of laser energy on the adhesion and wear resistance of the gradient coatings was analyzed. The results demonstrate that with increasing laser impact energy, the thickness of the AlCrN/TC4 transition layer gradually decreases from 3.75 μm to 1.32 μm, accompanied by significant changes in elemental distribution across the surface and cross-section. The interfacial bonding strength of the gradient coating increases substantially from 13.6 N to 43.3 N, while the average friction coefficient rises from 0.436 to 0.507. Concurrently, the wear track depth is reduced, and the wear rate decreases from 86.46 × 10^−5^ mm^3^/(N·m) to 7.67 × 10^−5^ mm^3^/(N·m). Laser shock peening promotes elemental diffusion, enabling the formation of a diffusion-aided interlayer. The incorporation of this diffused zone facilitates the successful construction of a high-quality TC4 titanium alloy gradient coating, effectively broadening the film–substrate interface, enhancing surface hardness, and significantly improving both interfacial adhesion and wear resistance.

## 1. Introduction

Titanium alloys, with their low density, high specific strength, and excellent corrosion resistance, are widely used in aerospace, marine engineering, and automotive manufacturing. However, their inherent drawbacks—low hardness and poor wear resistance—require pairing with PVD (Physical Vapor Deposition) wear-resistant coatings (such as CrAlN, MCrAlY, DLC, etc.) to withstand environments involving combined corrosion and wear [1,2,3,4]. However, lattice mismatch between the coating and titanium substrate, along with interfacial heterogeneity, often leads to residual tensile stresses and pinholes during coating deposition. These defects result in poor adhesion to the substrate. In the harsh, multi-field coupled environment of deep-sea operations, seawater readily infiltrates through inherent defects like pores, compromising the coating/substrate interface. This causes coating cracking and spalling, undermining the intended coating reinforcement effect [5,6,7]. Therefore, enhancing the interfacial adhesion and friction/wear resistance of AlCrN coatings is crucial for fully realizing their service performance.

Sandblasting and shot peening, as conventional post-treatment techniques for coatings, utilize compressed air as the driving force to propel abrasive particles or shot media onto the workpiece surface for surface modification [8]. Micro-sandblasting effectively reduces the number of surface droplets, thereby removing particulate contaminants from the coating and improving surface quality [9]. Both processes can alter the stress state of the coating or substrate, leading to enhanced mechanical performance [10]. However, shot peening often induces a significant deterioration in surface roughness [11]. Moreover, due to variations in interfacial properties such as thermal expansion coefficients and wetting behavior across different material systems, these mechanical methods are unable to tailor the composition and microstructure of the film–substrate interface or mitigate inherent material heterogeneity. Laser shock peening (LSP), while sharing certain functional similarities with shot peening and sandblasting, represents a more advanced surface modification technology. It offers distinct advantages in surface hardening, residual stress generation, and refinement of crystalline structures. LSP is frequently employed as a pretreatment method. For instance, Cao et al. [12] applied LSP to TC4 titanium alloy substrates prior to depositing multilayer gradient TiN/Ti coatings. Their results demonstrated a significant increase in surface hardness and compressive residual stress. Furthermore, LSP reduced interfacial deformation mismatch, suppressed fatigue crack initiation, and consequently improved fatigue resistance. Tang et al. [13] subjected Cr/CrN composite coatings on Zr alloy surfaces to laser shock peening. The results indicated that moderate laser energy effectively enhanced coating hardness and residual compressive stress while reducing wear rates by approximately 32%. Similarly, Sarvesh et al. [14] deposited AlTiN and AlCrN coatings on WC/Co substrates following LSP treatment. Friction tests revealed that coatings on laser-treated surfaces exhibited lower friction coefficients and reduced wear loss. However, that study employed only a water confinement layer, resulting in limited substrate ablation and insufficient plasma activation. The formation of carbon-deficient phases during ablation introduced physical mismatches at the interface, leading to poor coating adhesion. Collectively, these studies illustrate the application of surface treatments at both pre- and post-deposition stages. While conventional shot peening fails to engineer the film–substrate interface to resolve material incompatibility, LSP improves surface morphology. Nevertheless, the potential of laser-induced plastic deformation and thermal effects remains underexploited, and the role of elemental diffusion driven by micro-defect generation and plasma-mediated surface activation has not been thoroughly investigated.

This study investigates the effects of laser shock treatment on element diffusion within AlCrN/TC4 systems. It analyzes how element diffusion influences the secondary deposition of AlCrN coatings and evaluates the impact of laser shock treatment at different energy levels on the adhesion strength at the film–substrate interface and wear performance, focusing on coating composition and structural optimization.

## 2. Experimental Details

### 2.1. Preparation of Samples

**Figure 1:**
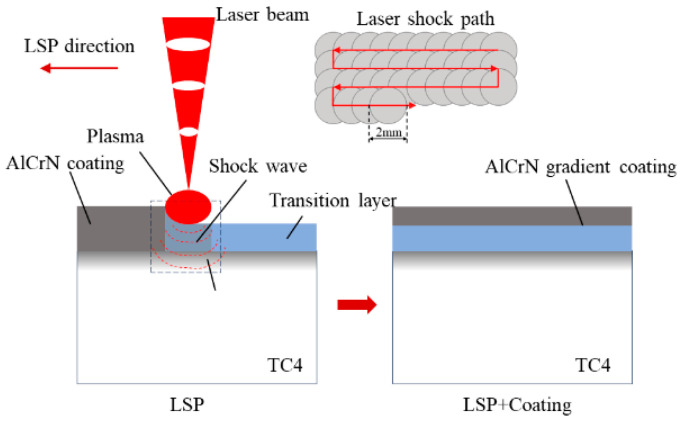
The preparation process of gradient coatings for elemental diffusion of LSP.

The fabrication process of the laser shock-induced gradient coating is illustrated in Figure 1. The substrate material is TC4 titanium alloy. The TC4 titanium alloy was processed into 30 mm × 30 mm × 5 mm specimens using wire cutting. Subsequently, the specimens were progressively polished with 500-grit, 1000-grit, and 2000-grit sandpaper. Polishing was performed using 0.5 μm diamond polishing compound. The specimens were then ultrasonically cleaned in high-purity anhydrous ethanol for 15 min and air-dried.

First, an AlCrN coating was deposited onto a titanium alloy substrate using a multi-arc ion plating machine (Wuxi Nanoarc New Materials Technology Co., Ltd., Wuxi, China, model NANOARC-SP1010). The substrate material was secured to a rotating fixture operating at 20 rpm. During the coating pretreatment stage, the chamber was first evacuated to 4.0 Pa, followed by a fine vacuum to 4.0 × 10^−3^ Pa. The vacuum chamber was heated to 80 °C. During the subsequent surface activation process, argon gas was introduced at a flow rate of 400 mL/min. A pulsed bias voltage of 600 V with a 50% duty cycle was applied. The TC4 titanium alloy surface was cleaned using argon ion glow discharge. During the coating deposition stage, 99.99% pure Cr and Al targets were used with a pulsed bias voltage of 150 V and a 15% duty cycle. The gas pressure was controlled at 0.8 Pa, arc current of 100 A, with nitrogen gas introduced at a flow rate of 400 mL/min. The Cr target was fully activated for 6 min of deposition, followed by 6 min with 50% target area activated, then fully activated for 6 min, and finally 50% activated for 12 min. Simultaneously, the Al target was opened at 50% area for 16 min of deposition, fully opened for 14 min, totaling 30 min of coating deposition to form a Cr-Al multilayer coating structure.

Second, an Nd:YAG laser was employed for elemental diffusion treatment of the AlCrN coating. The laser utilized a collimated-focused composite system with a 2 mm spot diameter, exhibiting a Gaussian spatial energy distribution at 1064 nm wavelength. pulse width of 9 ns, frequency of 50 Hz, and spot overlap ratio of 50%. A 1 mm thick optical glass was used as the constraint layer. Direct surface treatment was performed on the coating using laser energies of 0 J, 0.5 J, 1 J, and 1.5 J. Finally, AlCrN coating deposition was performed again to complete the establishment of the gradient coating. The schematic diagram of the laser shock element diffusion treatment and gradient coating preparation process is shown in Figure 1.

### 2.2. Microstructure Observation and Performance Testing

The surface and cross-sectional microstructural features of the AlCrN elemental diffusion layer subjected to laser shock peening at various energy levels were characterized using scanning electron microscopy (SEM, JSM-7200F, JEOL, Tokyo, Japan). Coating thickness was simultaneously measured during imaging. Elemental distribution across the surface and cross-section of the diffusion layer was analyzed via energy dispersive X-ray spectroscopy (EDS, Oxford X-Max, Oxford Instruments, Oxford, UK). The three-dimensional surface topography of the elemental diffusion gradient coating was evaluated using a confocal laser scanning microscope (μsurf mobile, Nano Focus, Oberhausen, Germany), and geometric parameters of the surface morphology were extracted using the integrated μsurf post-processing software.

Interfacial engineering adhesion strength, both before and after re-deposition of the coating, was assessed by means of an automatic scratch tester (WS-2005, Lanzhou Zhongke Kaihua Technology Development Co., Ltd., Lanzhou, China). A linearly increasing load from 0 to 60 N was applied over a scratch length of 4 mm at a loading rate of 60 N/min. Dry reciprocating sliding friction tests were performed on a tribometer (MFT-5000, Rtec Instruments Inc., San Jose, CA, USA) under ambient conditions. The evolution of normal load, friction force, and coefficient of friction was continuously recorded throughout the test. A constant normal load of 10 N was applied, with a total duration of 1800 s, a reciprocating stroke of 5 mm, and a frequency of 2 Hz. The lower specimen remained stationary while the upper counterpart executed linear reciprocating motion.

## 3. Results and Discussion

### 3.1. Effect of Laser Energy on the Surface Morphology and Elemental Distribution of AlCrN Coatings

**Figure 2:**
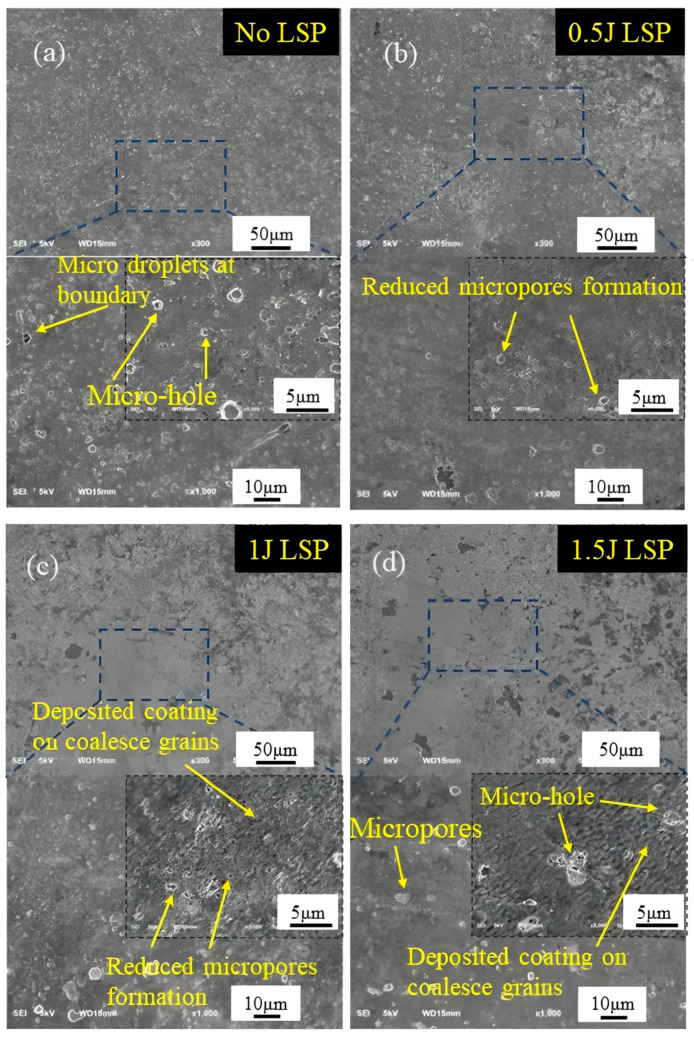
SEM microstructure of surfaces treated with LSP at different LSP energies: (**a**) No LSP; (**b**) 0.5 J LSP; (**c**) 1 J LSP; (**d**) 1.5 J LSP.

Figure 2a presents the initial surface microstructure of the AlCrN coating. As shown in Figure 2a, the surface exhibits inherent micro-defects such as micro-pores and micro-droplets, which are commonly observed in coatings fabricated by physical vapor deposition (PVD) [15,16,17]. These defects typically have diameters less than 1 μm. Their presence leads to microstructural inhomogeneity, thereby compromising the overall performance of the coating to a certain extent. Figure 2b–d display the surface morphologies of the AlCrN coating after laser shock peening (LSP) at energies of 0.5 J, 1 J, and 1.5 J, respectively. A significant reduction in the number of micro-defects, including micro-pores and micro-droplets, is observed, indicating that LSP effectively reduces particulate contamination on the PVD coating surface and exerts a cleaning effect on surface droplets. At lower energy levels, LSP does not impair the surface integrity of the AlCrN coating. However, in the absence of an absorption layer during treatment, higher laser energies induce intense shock waves that lead to plastic deformation and plasma formation, resulting in localized material accumulation, the formation of pronounced cavities and micro-pores, and even partial delamination of the coating.

**Figure 3:**
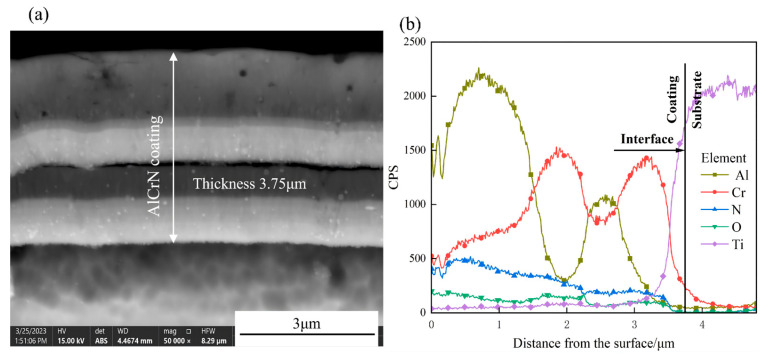
Test results for cross-sections: (**a**) SEM morphology of AlCrN coating; (**b**) element distribution of AlCrN coating.

Figure 3 shows the cross-sectional SEM image and EDS line-scan elemental distribution of the as-deposited AlCrN coating on the TC4 titanium alloy substrate. The initial coating surface is relatively flat, with an average thickness of approximately 3.75 μm, and exhibits a well-defined, continuous interface between the coating and substrate. Combined analysis of Figure 3a,b reveals a multi-layered architecture: the intermediate layer, primarily composed of Cr and Al, has a thickness of ~2.37 μm; the outermost Al-rich layer measures ~1.38 μm in thickness; and a thin Cr-rich interlayer (~0.6 μm) is present at the coating–substrate interface, facilitating adhesion to the TC4 substrate.

**Figure 4:**
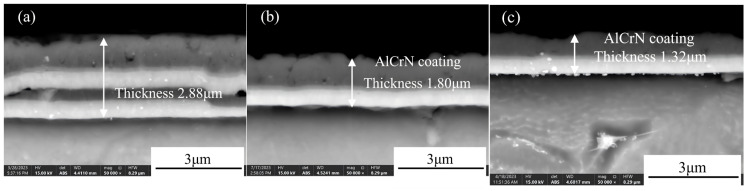
SEM morphology of AlCrN coating for cross-sections with different LSP energies: (**a**) 0.5 J LSP; (**b**) 1 J LSP; (**c**) 1.5 J LSP.

Figure 4 presents the cross-sectional SEM images of AlCrN coatings deposited on TC4 titanium alloy substrates after LSP at various energy levels. Compared to the as-deposited coating, increasing laser energy leads to a progressive reduction in coating thickness from 3.75 μm to 1.32 μm, accompanied by an intensification of surface micro-pitting and the development of irregular, tooth-like morphological features—characteristics consistent with multi-spot laser-induced surface plastic deformation [18]. At higher energies (1 J and 1.5 J), structural changes are observed in the intermediate layer of the AlCrN coating, including further compression of the overall coating thickness and evidence of interlayer compaction and fusion between constituent elemental layers.

**Figure 5:**
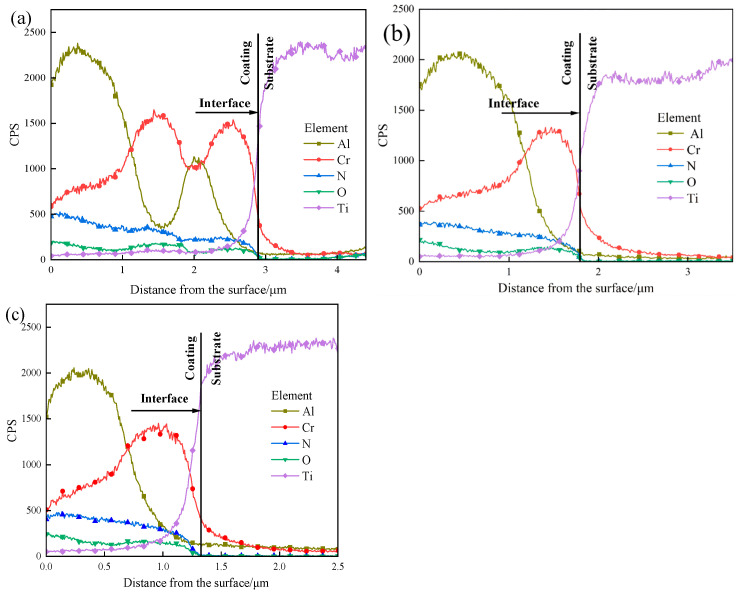
Element distribution of AlCrN coating for cross-sections with different LSP energies: (**a**) 0.5 J LSP; (**b**) 1 J LSP; (**c**) 1.5 J LSP.

Figure 5 displays the EDS line-scan profiles across the cross-sections of the coatings following LSP treatment. As shown in Figure 5a, after LSP at 0.5 J, the AlCrN coating retains its original multilayer architecture, consisting of an intermediate layer and a surface Al-rich layer. The total coating thickness is approximately 2.88 μm, with the surface Al layer measuring ~1.25 μm, the intermediate layer ~1.63 μm, and the Cr-rich interfacial layer adjacent to the TC4 substrate ~0.48 μm in thickness. In Figure 5b, following 1 J LSP treatment, the total coating thickness decreases to ~1.80 μm. The laser-induced compressive stress promotes lateral diffusion of Al atoms along the surface region, resulting in minimal change in the Al-layer thickness. However, the intermediate layer undergoes significant thinning (~0.97 μm reduction), with its composition shifting from ternary Al–Cr–N to binary Cr–N, indicating pronounced elemental redistribution and interdiffusion. Concurrently, the Cr-rich interfacial layer thickens to ~0.65 μm. This behavior arises from the differing responses of Al and Cr atoms to the shock wave: due to its higher hardness and chemical inertness, Cr exhibits greater resistance to displacement and preferentially migrates toward the substrate, forming a diffusion-bonded interface with the TC4 alloy. Laser shock peening introduces a high density of crystallographic defects such as dislocations, dislocation walls, and dislocation tangles. These defect-induced lattice distortions lower the activation barrier for atomic migration, thereby enhancing diffusivity and facilitating interlayer element diffusion [19].

As illustrated in Figure 5c, after LSP at 1.5 J, the overall coating thickness is further reduced. The intermediate layer remains predominantly composed of Cr and N, with elemental distribution patterns largely unchanged compared to the 1 J condition. The Cr-rich interfacial layer measures ~0.43 μm, comparable to its initial width prior to treatment. This stabilization is attributed to the fact that, at 1.5 J, the induced shock wave pressure exceeds 5 GPa (based on the laser shock pressure-time profile), driving the TC4 substrate into a state of maximum plastic deformation and peak microhardness. Under these extreme conditions, the resulting high-density dislocation network acts as a diffusion barrier, effectively impeding further Cr atom migration.

**Figure 6:**
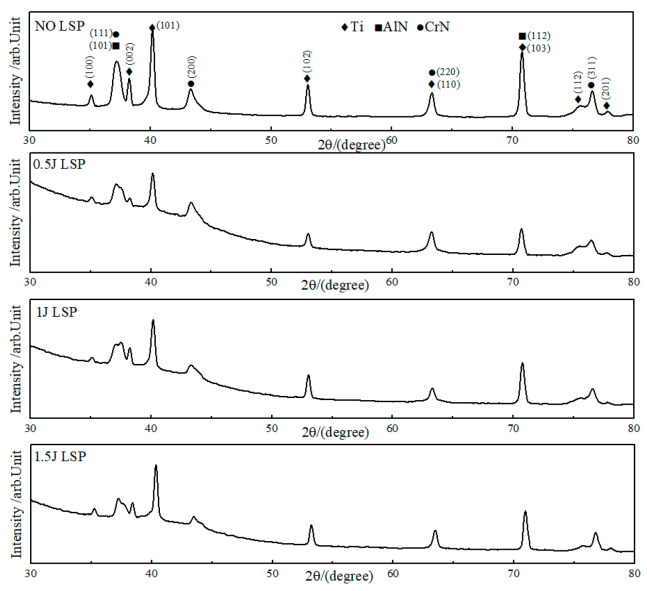
XRD patterns of LSP processing at different LSP energies.

Figure 6 presents the X-ray diffraction (XRD) patterns of the AlCrN coating surface following laser shock peening (LSP) at various energy levels. As demonstrated in the accompanying figure, following laser shock peening treatment, the diffraction peaks of the material invariably encompass (111), (200), (311), (100), (002), (101), (102), amongst others, thereby encompassing the primary phases of the AlCrN coating. The AlN phase and CrN phase, in addition to the primary phases of the TC4 titanium alloy, encompass the α-Ti phase and β-Ti phase. As the impact energy increases, no new diffraction peaks appear, indicating no phase transformation or new phase formation during this process. However, a significant decrease in the intensity of the characteristic diffraction peaks at (111), (200), and (311) is observed. This phenomenon is attributed to the reduction in AlCrN coating thickness following laser shock peening at different energies, resulting in a diminished volume of coating participating in diffraction. With increasing impact energy, the intensities of the Ti(101), (102), and (103) diffraction peaks exhibited an upward trend. This phenomenon is attributed to the refinement of the grains caused by the laser shock peening effect. Furthermore, the presence of deformation texture on these crystal planes during the LSP process may also contribute to the increased diffraction peak intensity [20].

**Figure 7:**
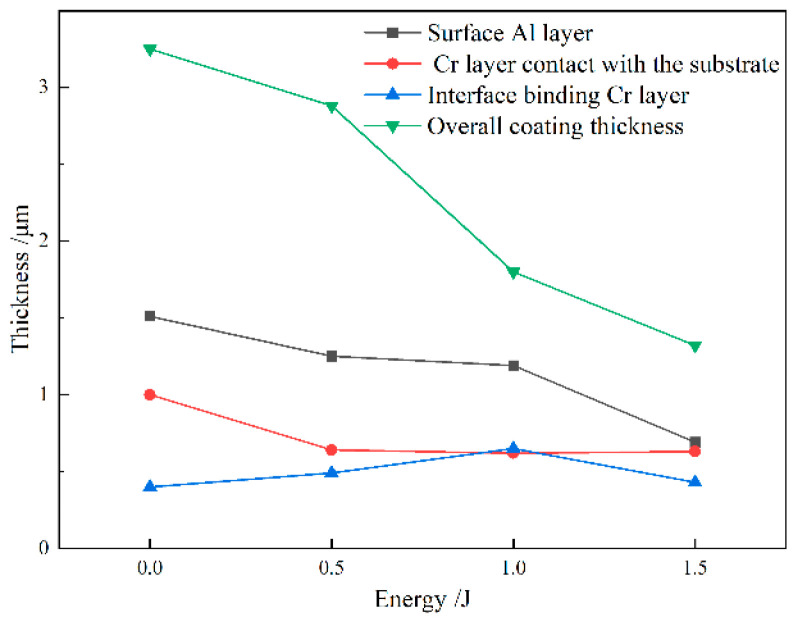
Thickness of each part of AlCrN coating with different LSP energies.

**Figure 8:**
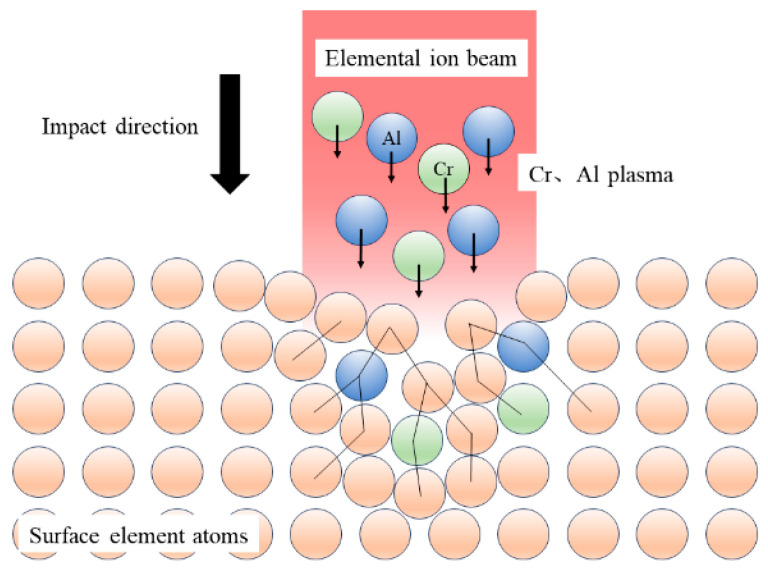
Schematic diagram of atomic diffusion of plasma impact elements.

Figure 7 presents the thickness variation in the AlCrN coating following laser shock peening (LSP) at different energy levels. As shown, the overall coating thickness progressively decreases with increasing laser energy, attributed to the combined effects of plasma-induced ablation and shock-induced plastic deformation. At 0.5 J, the thickness of all constituent layers is reduced, indicating uniform material compaction and surface erosion. Upon treatment at 1 J, the thicknesses of both the surface Al layer and the interfacial Cr layer exhibit minimal change. This stability arises from two concurrent mechanisms: first, a limited amount of Al atoms undergo lateral diffusion along the surface region following plasma formation, compensating for material loss; second, due to their distinct dynamic response under shock loading, Cr atoms preferentially migrate toward the substrate, resulting in interfacial broadening between the Cr layer and the TC4 alloy [21,22]. The maximum interfacial width is achieved at 1 J, suggesting optimal diffusion conditions at this energy level. A schematic illustration of the plasma-driven atomic diffusion and infiltration process is provided in Figure 8.

At 1.5 J, the higher laser energy intensifies plasma generation, leading to more pronounced surface material removal. Simultaneously, the induced shock pressure exceeds 5 GPa, driving the TC4 substrate into a state of maximum plastic deformation, where microstructural refinement and microhardness reach saturation. Under these extreme conditions, the high density of dislocations generated by intense shock loading acts as a barrier to long-range atomic diffusion, thereby suppressing further interfacial element migration. Consequently, the thickness of the Cr-rich bonding layer stabilizes and remains comparable to that of the as-deposited state.

### 3.2. Effect of Element Diffusion Layers Induced by Varying Laser Energies on the Performance of Gradient Coatings

#### 3.2.1. Surface Morphology and Hardness of the Gradient Coating

**Figure 9:**
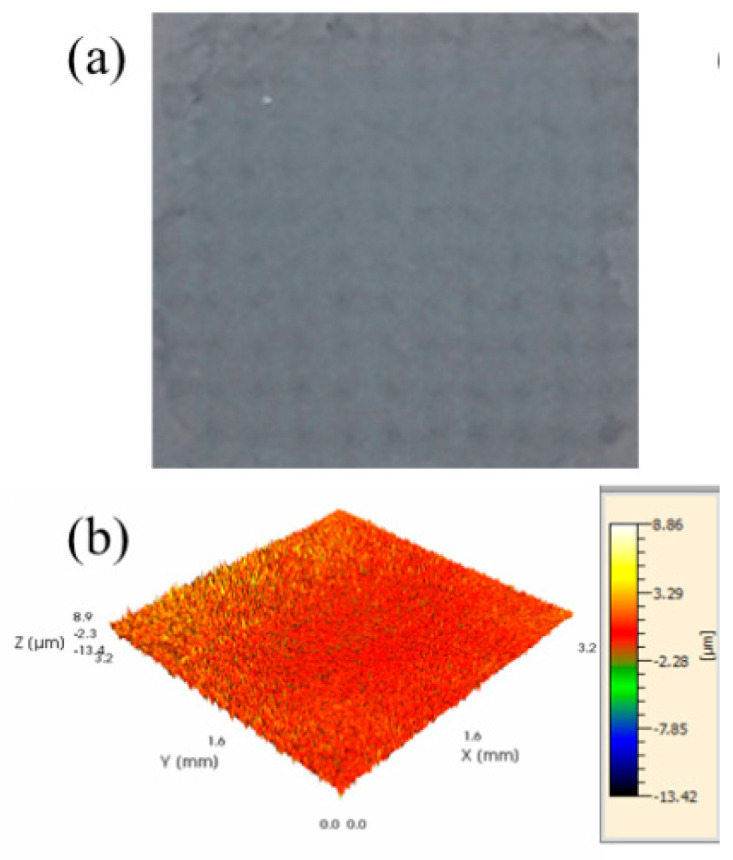

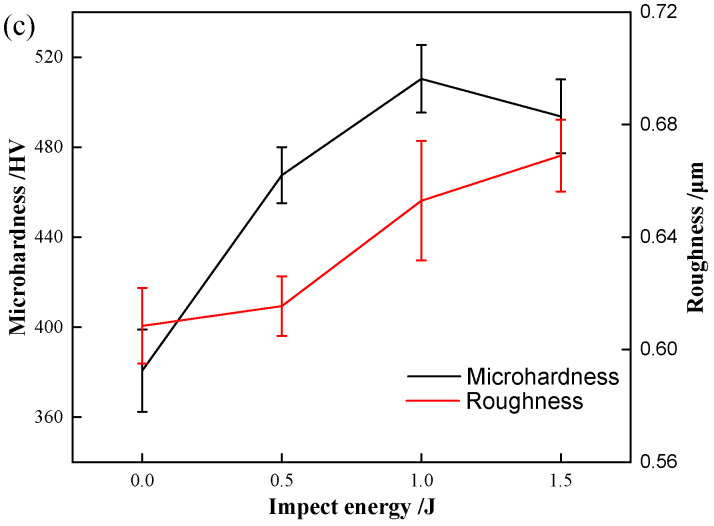
(**a**) Gradient coating of the surface; (**b**) three-dimensional topography of gradient coatings; (**c**) roughness and microhardness curves of gradient coatings with different LSP energies.

The surface and three-dimensional morphology of the gradient layer are presented in Figure 9a,b. The surface exhibits a gray appearance, with a clearly observable array of micro-pits. This morphological feature arises due to the micrometer-scale thickness of the post-deposited coating, while the initial AlCrN coating undergoes a thickness reduction of 2.43 μm under laser shock treatment—comparable to the thickness of the subsequently deposited layer. Figure 9c displays the surface roughness and microhardness of gradient coatings fabricated at different laser energies. With increasing shock energy, the surface roughness progressively increases from 0.609 μm to 0.669 μm, representing an approximate 10% rise. Concurrently, microhardness measurements indicate that all gradient coatings exhibit enhanced hardness relative to the as-deposited AlCrN coating. The average microhardness of the initial coating is 380.1 HV, whereas the gradient coatings achieve an average value of 490.6 HV. As the laser energy increases, the hardness enhancement reaches 22.9%, 34.2%, and 29.7%, respectively. It shows a trend of increasing first and then decreasing. This improvement is attributed to shock-induced grain refinement: during laser shock peening, the surface grains progressively refine, significantly increasing the density of grain boundaries and thereby enhancing resistance to plastic deformation [23,24]. The grains eventually reach the nanometer scale and form a nanocrystalline structure, beyond which further refinement is limited. Consequently, the surface microstructure becomes more homogeneous, leading to a macroscopic saturation in hardness.

#### 3.2.2. Effect of Varying Laser Energies on the Interfacial Bonding Performance of Gradient Coatings

**Figure 10:**
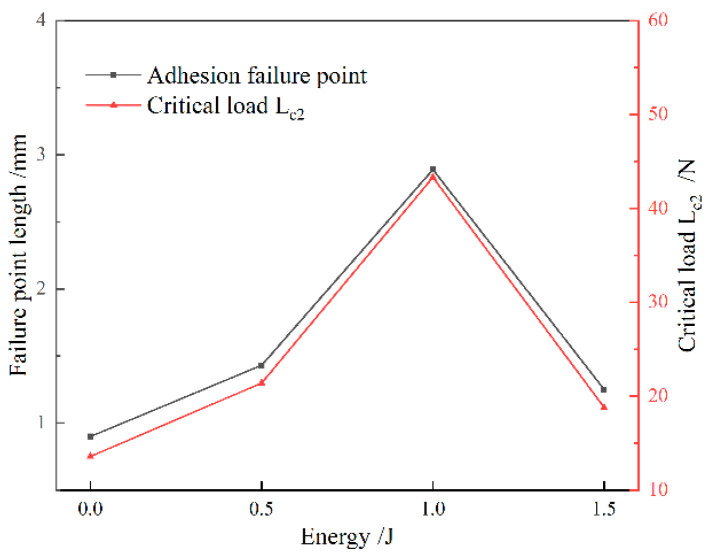
Bonding strength of gradient coatings fabricated using diffusion layers with varying energy levels.

**Figure 11:**
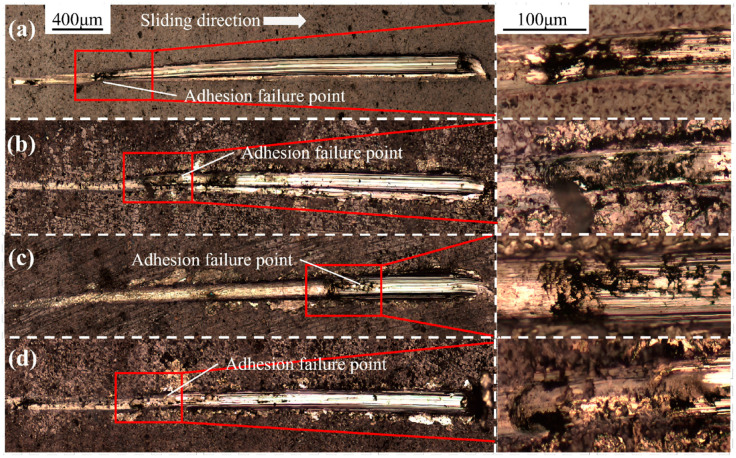
Scratch topography of gradient coating with different LSP energies: (**a**) No LSP; (**b**) 0.5 J LSP; (**c**) 1 J LSP; (**d**) 1.5 J LSP.

Figure 10 presents the bonding strength of gradient coatings fabricated using diffusion layers with varying energy levels. Figure 11 presents the scratch morphologies of gradient coatings fabricated under different laser energies, captured at magnifications of 100× and 200×. The engineering bonding strength was quantitatively evaluated using the critical load Lc2, with the corresponding positions and morphological features clearly marked. In the scratch test, three distinct failure stages are observed: the initiation of fine cracks (critical load Lc1), coating spallation (critical load Lc2), and complete delamination (critical load Lc3) [25]. The critical load Lc2 was adopted as the representative measure of engineering bonding strength. As shown in Figure 10, the initial AlCrN coating exhibits an engineering bonding strength of 13.6 N. With increasing laser shock energy, the bonding strength of the element-diffusion-based gradient coatings first increases and then slightly decreases, with all values exceeding that of the initial coating. The maximum bonding strength of 43.3 N is achieved at 1 J, significantly higher than the baseline value of 13.6 N. At 0.5 J and 1.5 J, the measured strengths are 21.4 N and 18.8 N, respectively. Under the action of laser-induced shock waves, plastic deformation and plasma generation alter the elemental distribution, promoting interdiffusion between coating and substrate atoms. This process increases the interfacial width, while lattice distortions caused by microscale defects facilitate atomic diffusion and migration. Elemental interdiffusion not only strengthens the interface through the formation of diffusion-bonded junctions but also enhances compatibility by homogenizing thermal expansion coefficients, reducing material heterogeneity, and relieving residual stresses at the film–substrate interface, thereby improving interfacial adhesion. Additionally, laser shock treatment increases the surface roughness of the substrate, enabling mechanical interlocking between the coating and the textured surface—commonly described as a “hook-and-loop” or anchoring effect—which enlarges the effective contact area and enhances mechanical coupling [26]. However, excessive laser energy induces stress concentration at the interface, which may initiate microcracks and limit further improvement in bonding performance [27]. Furthermore, the plasma generated during laser shock processing activates the substrate surface, increases its surface energy, and improves wettability, thereby promoting better coating adhesion and facilitating the formation of a graded interface—another contributing factor to enhanced bonding strength [28].

#### 3.2.3. Effect of Laser Shock Energy on the Friction and Wear Behavior of Gradient Coatings with Element Diffusion 

**Figure 12:**
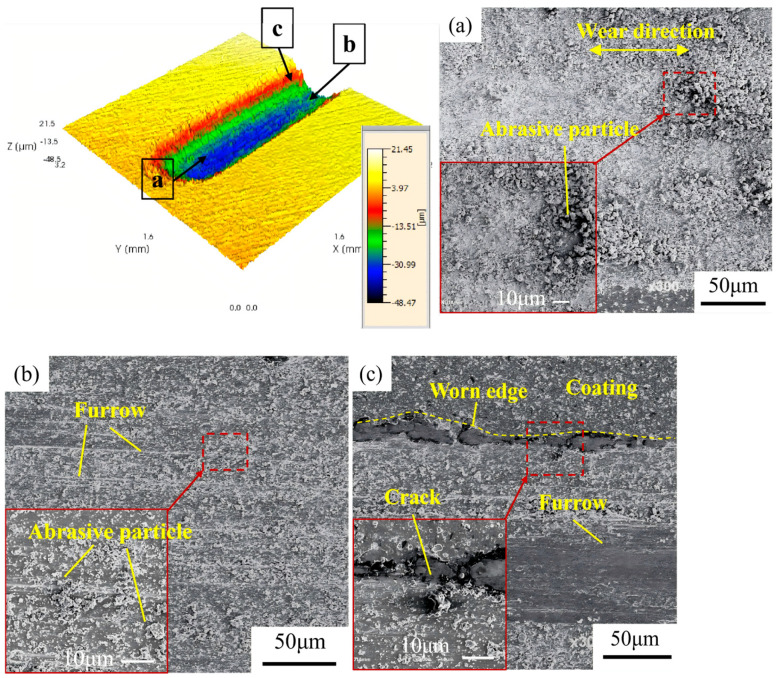
Friction and wear morphology of the initial AlCrN coating: (**a**) oval contact zone; (**b**) center contact zone; (**c**) the edge of the contact area.

Figure 12 presents the friction and wear morphology analysis of the initial AlCrN coating, including the three-dimensional topography obtained by confocal laser scanning microscopy (CLSM) and the corresponding SEM micrographs. The average wear depth of the initial AlCrN coating is approximately 47.86 μm. Figure 12a depicts an elliptical contact zone located at the reversal ends of the reciprocating motion, where plastic strain energy accumulates. Severe accumulation of abrasive particles is observed in this region, which elevates the local surface and results in predominant material loss occurring within the built-up debris layer. Figure 12b illustrates the central contact region, characterized by uniformly distributed grooves formed through ploughing action, along with a significant presence of blocky wear debris. Figure 12c shows the periphery of the contact area, where the morphology is complex and consists of coating cracks, wear particles, ploughing furrows, and pitting spallation. Pronounced coating delamination is evident, leading to substrate exposure and the initiation of fatigue wear.

**Figure 13:**
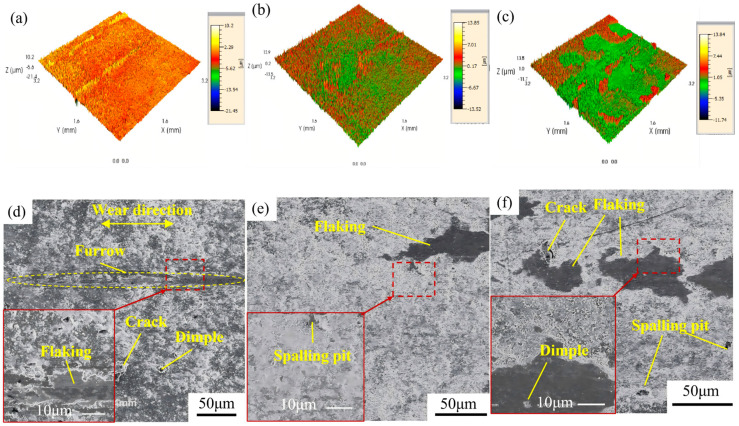
Friction and wear morphology of gradient coatings with different LSP energies: (**a**,**d**) 0.5 J; (**b**,**e**) 1 J; (**c**,**f**) 1.5 J.

Figure 13 displays the friction and wear morphologies of gradient coatings fabricated under different laser energies. Comparative analysis of the three-dimensional profiles in Figure 12 and Figure 13 reveals that the gradient coatings exhibit significantly reduced wear depths compared to the initial AlCrN coating under identical tribological conditions. As shown in Figure 13a,d, for the gradient coating processed at 0.5 J, features such as abrasive particles, microcracks, and localized spallation are observable. The coating is damaged in the central contact zone, resulting in substrate exposure and the formation of a groove approximately 10 μm in depth. Limited spallation and cracking are present around the central groove, indicating a wear mechanism dominated by abrasive and fatigue wear. In Figure 13b,e, the gradient coating treated at 1 J exhibits blocky and laminar delamination, with abundant abrasive particle distribution. These particles act as a protective interlayer, preventing direct metal-to-metal contact between the sliding counterparts, thereby confining material removal to the debris layer and effectively protecting the underlying substrate. The dominant wear mechanism is abrasive wear. For the gradient coating prepared at 1.5 J, as seen in Figure 13c,f, in addition to abrasive debris, extensive spallation is observed on the surface. Pits and microcracks are present on the exposed substrate and its surrounding areas, suggesting combined effects of abrasive wear and subsurface fatigue, consistent with a mixed wear mechanism involving both abrasion and fatigue-induced delamination.

**Figure 14:**
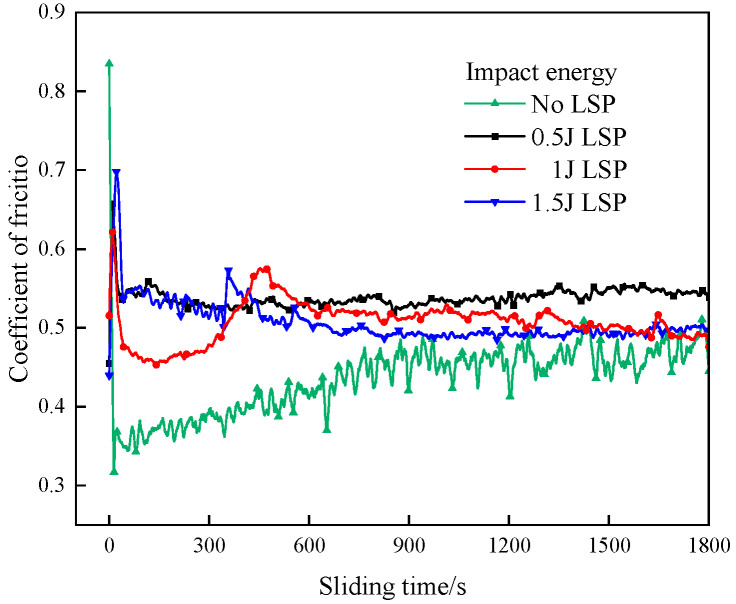
Friction coefficient variation curve with sliding time with different LSP energies.

Figure 14 presents the evolution of friction coefficients as a function of sliding time for gradient coatings fabricated under different laser energies. The initial AlCrN coating exhibits an average friction coefficient of 0.434, which gradually increases with sliding time, accompanied by an escalating fluctuation amplitude. This trend is attributed to progressive coating delamination, leading to substrate exposure and direct contact between the counterface and the mixed coating–substrate interface, thereby increasing frictional resistance. For the gradient coating processed at 0.5 J, the average friction coefficient rises to 0.537, and the curve remains consistently elevated throughout the test. This indicates limited improvement in anti-fatigue wear performance, consistent with the observed morphological features such as pronounced ploughing grooves, microcracks, and localized spallation. The gradient coating treated at 1 J shows an average friction coefficient of 0.507. Its friction curve initially decreases—likely due to surface conditioning—followed by a transient increase and subsequent reduction before stabilizing. At approximately 460 s, noticeable wear develops, signifying that the coating’s self-lubricating and wear-resistant capacity has reached its limit, resulting in the onset of abrasive wear. Nevertheless, owing to its superior surface hardness and interfacial bonding strength, the coating maintains a relatively stable friction response with minimal fluctuations. The gradient coating fabricated at 1.5 J has an average friction coefficient of 0.505. In the early stage of sliding, its friction behavior resembles that of the 0.5 J sample. However, at around 340 s, a sharp fluctuation in the friction coefficient occurs, indicating severe degradation of surface integrity. This instability coincides with extensive coating spallation and the initiation of mild fatigue wear on the exposed substrate. Following this transition period, the friction coefficient gradually stabilizes, suggesting the establishment of a steady wear regime.

**Figure 15:**
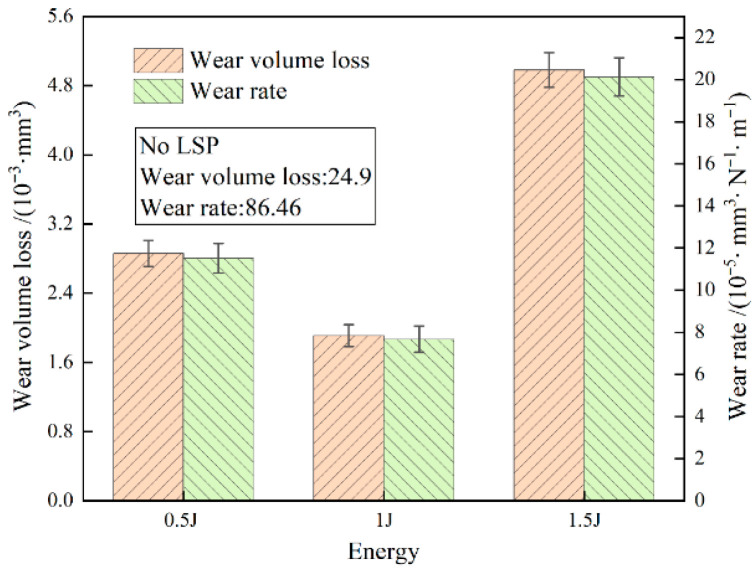
Wear volume loss and wear rate with different LSP energies.

Figure 15 presents the wear rates of gradient coatings fabricated under different laser shock energies. The initial AlCrN coating exhibits a wear volume of 24.9 × 10^−3^ mm^3^ and a wear rate of 86.46 × 10^−5^ mm^3^/(N·m), both significantly higher than those of the gradient-coated samples. With increasing laser energy, the wear rates of the gradient coatings first decrease and then increase, with all values remaining substantially lower than that of the initial AlCrN coating. The minimum wear volume (1.91 × 10^−3^ mm^3^) and wear rate (7.67 × 10^−5^ mm^3^/(N·m)) are achieved at an impact energy of 1 J, indicating optimal tribological performance at this energy level. The formation of the gradient structure leads to a significant increase in surface microhardness and improved interfacial adhesion strength. As a result, both wear rate and wear volume are markedly reduced during the friction and wear process, demonstrating a substantial enhancement in wear resistance compared to the pristine AlCrN coating. The gradient architecture alters the wear mechanism from severe abrasive wear and substrate fatigue damage to mild abrasive wear occurring predominantly on the coating surface. Additionally, wear debris generated from the gradient layer tends to accumulate, forming a protective debris layer. In subsequent sliding cycles, material removal primarily occurs within this layer, thereby preventing direct loss of the underlying metallic substrate. The improvements can be attributed to laser shock peening (LSP): on one hand, LSP induces grain refinement, enhances material hardness, and effectively inhibits crack initiation and propagation; on the other hand, it promotes elemental interdiffusion at the interface, increasing the interfacial width and reducing material heterogeneity. This results in a compositionally graded transition zone with gradually varying elemental distribution, which strengthens interfacial bonding and significantly suppresses coating spallation [21,22].

## 4. Conclusions

Gradient coatings with elemental diffusion were successfully fabricated by applying laser shock peening (LSP) at different energy levels to AlCrN-coated substrates. The influence of laser energy on the surface and cross-sectional morphology, as well as elemental distribution, was systematically investigated. The elemental diffusion behavior induced by the LSP process was analyzed, and the effects of the resulting gradient structure on interfacial bonding strength and tribological performance were evaluated. The following conclusions are drawn:

(1) Laser shock peening effectively mitigates inherent surface defects in AlCrN coatings, such as microdroplets and micropores. It promotes significant redistribution of alloying elements: aluminum (Al) migrates toward the surface layer, while chromium (Cr) diffuses inward. This interdiffusion broadens the interface between coating layers and establishes a compositionally graded structure, confirming the occurrence of pronounced elemental diffusion.
(2) The surface roughness of the element diffusion gradient coating is slightly higher than that of the initial AlCrN coating. The average microhardness value increased from 380.1 HV to 490.6 HV. The hardness values of gradient coatings established at different energies increased by 22.9%, 34.2%, and 29.7%, respectively, exhibiting a trend of first increasing and then decreasing. The interface engineering strength of the coating first increased and then slightly decreased, rising from a maximum of 13.6 N to 43.3 N, indicating a significant improvement in bonding performance.
(3) Compared to the original AlCrN coating, the element-diffusion gradient coatings exhibit a higher average friction coefficient (increasing from 0.436 to 0.537), yet demonstrate significantly reduced wear scar depth and a dramatic decrease in wear rate—from 86.46 × 10^−5^ mm^3^/(N·m) to 7.67 × 10^−5^ mm^3^/(N·m). This indicates a substantial enhancement in wear resistance, despite the elevated friction level, suggesting a transition in wear mechanisms toward milder surface degradation and effective protection of the substrate.

## Data Availability

The original contributions presented in this study are included in the article. Further inquiries can be directed to the corresponding author.
